# NR4A1 deletion promotes pro-angiogenic polarization of macrophages derived from classical monocytes in a mouse model of neovascular age-related macular degeneration

**DOI:** 10.1186/s12974-023-02928-1

**Published:** 2023-10-19

**Authors:** Steven Droho, Andrew P. Voigt, Jacob K. Sterling, Amrita Rajesh, Kyle S. Chan, Carla M. Cuda, Harris Perlman, Jeremy A. Lavine

**Affiliations:** 1 Department of Ophthalmology, Feinberg School of Medicine, Northwestern University, Chicago, IL 60611 USA; 2 Division of Rheumatology, Department of Medicine, Feinberg School of Medicine, Northwestern University, Chicago, IL 60611 USA

**Keywords:** Angiogenesis, Choroidal neovascularization, Macrophage, Monocyte, Non-classical monocytes, Neovascular age-related macular degeneration

## Abstract

**Background:**

Neovascular age-related macular degeneration causes vision loss from destructive angiogenesis, termed choroidal neovascularization (CNV). *Cx3cr1*^*−/−*^ mice display alterations in non-classical monocytes and microglia with increased CNV size, suggesting that non-classical monocytes may inhibit CNV formation. NR4A1 is a transcription factor that is necessary for maturation of non-classical monocytes from classical monocytes. While *Nr4a1*^*−/−*^ mice are deficient in non-classical monocytes, results are confounded by macrophage hyper-activation. *Nr4a1*^*se2/se2*^ mice lack a transcriptional activator, resulting in non-classical monocyte loss without macrophage hyper-activation.

**Main body:**

We subjected *Nr4a1*^*−/−*^ and *Nr4a1*^*se2/se2*^ mice to the laser-induced CNV model and performed multi-parameter flow cytometry. We found that both models lack non-classical monocytes, but only *Nr4a1*^*−/−*^ mice displayed increased CNV area. Additionally, CD11c^+^ macrophages were increased in *Nr4a1*^*−/−*^ mice. Single-cell transcriptomic analysis uncovered that CD11c^+^ macrophages were enriched from *Nr4a1*^*−/−*^ mice and expressed a pro-angiogenic transcriptomic profile that was disparate from prior reports of macrophage hyper-activation.

**Conclusions:**

These results suggest that non-classical monocytes are dispensable during CNV, and NR4A1 deficiency results in increased recruitment of pro-angiogenic macrophages.

**Supplementary Information:**

The online version contains supplementary material available at 10.1186/s12974-023-02928-1.

## Background

Age-related macular degeneration (AMD) is one of the leading causes of blindness in the developed world. AMD is first diagnosed when yellow, inflammatory lipoprotein deposits called drusen develop underneath the retinal pigment epithelium (RPE). Drusen, which are composed of many factors including lipids and complement proteins [1], create a chronic, inflammatory microenvironment between the RPE and choriocapillaris, which nourishes the RPE. This complement-driven inflammatory microenvironment stimulates monocyte and macrophage recruitment, which can lead to pathological and destructive angiogenesis [2], termed choroidal neovascularization (CNV), which is the key feature of neovascular AMD. Neovascular AMD is currently treated with anti-vascular endothelial growth factor (VEGF) medications which improve vision by 5–10 letters on average [3, 4]. Despite maximal monthly anti-VEGF treatment, however, 15% of patients still lose vision [5]. Thus, an unmet need exists for anti-VEGF-resistant neovascular AMD patients.

Since drusen include complement factors that recruit macrophages [1], macrophages express both complement components and receptors [6], and AMD is associated with many complement genes [7–9], macrophages are a potential target for anti-VEGF resistant patients. In support of this hypothesis, surgically excised CNV membranes contain macrophages [10], and global macrophage ablation decreases laser-induced CNV, a murine model of neovascular AMD [11, 12]. However, macrophages are highly heterogeneous cells, displaying diverse transcriptional signatures [13], and their function is based upon both their origin and microenvironment. Choroidal macrophages are known to be fully replenished from the monocyte pool [14], which includes both classical and non-classical monocytes. *Ccr2*^*−/−*^ mice, which are deficient in classical monocyte recruitment to tissue, show decreased CNV area equivalent to global macrophage ablation [15–17], suggesting that classical monocytes and classical monocyte-derived macrophages (MDMs) are pathogenic during CNV. Furthermore, our lab has previously shown that CD11c^+^ macrophages are derived from classical monocytes, express a pro-angiogenic transcriptome in both mice and humans, and are necessary for the laser-induced CNV model [16, 18]. However, little is known about the role of non-classical monocytes and non-classical MDMs in the laser-induced CNV model.

Non-classical monocytes express high levels of CD43 and Cx3cr1, and low levels of CCR2 and Ly6C [19]. Lack of Cx3cr1 expression in non-classical monocytes impairs their function, including their ability to stimulate endothelial cell proliferation and regeneration after carotid injury [20]. Furthermore, the *Cx3cr1*^*−/−*^ mouse, which demonstrates both non-classical monocyte dysfunction and microglial alterations, shows increased CNV area [21]. These data suggest that non-classical monocytes may suppress pathological angiogenesis during laser-induced CNV. In agreement with a pathogenic role for classical monocytes and homeostatic role of non-classical monocytes in the eye, *Ccr2*^*−/−*^ and *Ccl2*^*−/−*^ mice are resistant to diabetic retinopathy [22, 23], while *Cx3cr1*^*−/−*^ and *Nr4a1*^*−/−*^ mice, two models of non-classical monocyte deficiency, show exacerbated diabetic retinopathy progression [24, 25]. NR4A1 is transcription factor that is necessary for maturation of non-classical monocytes from classical monocytes [26]. Although *Nr4a1*^*−/−*^ mice lack non-classical monocytes, NR4A1 loss can lead to macrophage hyper-activation [27], confounding results from this model. To address this limitation, *Nr4a1*^*se2/se2*^ mice lack a super enhancer 2 region located 4 kb upstream of the *Nr4a1* transcriptional start site and display non-classical monocyte deficiency without macrophage hyper-activation [28].

To determine the role of NR4A1 and non-classical monocytes in the laser-induced CNV model, we performed multi-parameter flow cytometry and measured CNV size in both *Nr4a1*^*−/−*^ and *Nr4a1*^*se2/se2*^ mice. We found that both models were deficient in non-classical monocytes, but CNV area was increased in only *Nr4a1*^*−/−*^ mice. Additionally, CD11c^+^ macrophage numbers were increased from *Nr4a1*^*−/−*^ laser-treated eyes. Next, we performed single-cell transcriptional profiling and found that a CD11c^+^ macrophage subtype was enriched from *Nr4a1*^*−/−*^ laser-treated eyes and expressed a pro-angiogenic transcriptome. These data suggest that non-classical monocytes are dispensable in the laser-induced CNV model and that *Nr4a1* deficiency alters gene expression toward a more pro-angiogenic transcriptional profile.

## Methods

### Animals

All studies were performed on 10- to 12-week-old mice. Wildtype (C57BL/6J; stock #000664), *Nr4a1*^*−/−*^ (B6;129S2-*Nr4a1*^*tm1Jmi*^/J; #006187) and *Nr4a1*^*se2/se2*^ (C57BL/6-*Rr39*^*em1Ched*^/J; #030204) animals were bred in house for one or two generations from breeders purchased from The Jackson Laboratory (Bar Harbor, ME). All experiments were approved by the Northwestern University Institutional Animal Care and Use Committee, and adhere to the ARVO statement for the Use of Animals in Ophthalmic and Vision Research. Genotyping was confirmed by Transnetyx, Inc. (Cordova, TN).

### Laser-induced CNV model

Experiments were performed as previously described [29]. Briefly, anesthetized mice had their eyes dilated and lasered with a slit lamp (Zeiss) mounted ophthalmic laser (IRIDEX). Each eye was treated with either 4 (CNV area quantification) or 8 (scRNA/CITE-Seq and flow cytometry analysis) focal laser burns (75 μm, 110 mW, 100 ms).

### Multi-parameter flow cytometry analysis

Experiments were performed as described [18] on female mice. Posterior eye cups were prepared into single cell suspensions and treated according to the detailed flow cytometry protocol [30]. Antibodies for preparation and single color controls can be found in Additional file 7: Table S5. Samples were run on a BD FACSSymphomy A5-Laser Analyzer (BD Biosciences) at the Northwestern University RHLCCC Flow Cytometry Facility.

### Immunofluorescence staining

Enucleated eyes were stained as previously described [29]. Choroidal whole mounts (RPE, choroid, and sclera) were fixed and stained with the antibodies described in Additional file 7: Table S5. Images were obtained on a Nikon Ti2 Widefield microscope using a 10 × objective in the Northwestern Center for Advanced Microscopy using Nikon NIS Elements software (Nikon). ImageJ software (NIH) was used to measure CNV area by a masked grader.

### Ex vivo* choroidal sprouting assay*

Experiments were performed as described [29] on female mice. Pieces of choroid were dissected and placed on Matrigel (Corning) in endothelial cell growth media (EGM2, Lonza). Media were changed every 2 days and pictures were taken on day 4 with a Nikon Ti2 Widefield microscope using a 4 × objective and Nikon NIS Elements software. Images were analyzed with Nikon Elements General Analysis. Images were preprocessed with edge detection and segmented with thresholding. Total area was measured for each image with central choroidal tissue subtracted from the angiogenesis area.

### Single-cell RNA-seq and CITE-Seq sample preparation

Single-cell suspensions of posterior eye cups were obtained as previously described [18] using female mice. Prior to any staining, cell suspensions were subjected to a CD45 enrichment using the protocol provided by the manufacturer (Miltenyi Biotec, 130-052-301). Enriched, live cells were stained with ADT antibodies (Additional file 8: Table S6), CD45, and CD11b (Additional file 7: Table S5) and sorted on a BD FACSAria cell sorter (BD Biosciences) at the Northwestern University RHLCCC Flow Cytometry Facility. Sorted populations were collected in MACS buffer (Miltenyi Biotec, 130-091-221). Cells were centrifuged at 350×*g *for 10 min at 4 °C and resuspended in RPMI (MilliporeSigma, R8758). scRNA-seq libraries were prepared as previously described [31] using the Single Cell 3’ v3 Reagent Kit. Gel Bead in emulsions were generated by the 10 × Genomics Chromium Controller in the Northwestern Metabolomics Core Facility. Barcoded libraries were sequenced on the NextSeq 2000 platform using the P3 flow cell 100 bp. FASTQ files were generated from basecalls using bcl2fastq (Illumina). Resulting FASTQ files were mapped to the mouse genome mm10 using CellRanger v7.0.1 (10X Genomics, Pleasanton, CA) and demultiplexed into independent RNA and antibody derived tag (ADT) libraries.

### Bioinformatics: clustering

To remove low-quality cells and doublets, cells were filtered if they had fewer than 200 unique RNA features, greater than 5000 unique RNA features, or had greater than 15% of RNA reads mapping to the mitochondrial genome. After filtering, we removed 401 cells, and 11,223 (96.55%) cells remained for downstream analysis (*n* = 3791 WT-laser, *n* = 1649 *Nr4a1*-laser, *n* = 3763 WT-control, *n* = 2020 *Nr4a1*-control). Filtered RNA and ADT assays were independently normalized, scaled, and integrated with reciprocal principal component analysis [32] using the Seurat R package (version 4.1) [33]. Integrated RNA and ADT assays were then combined with weighted nearest neighbor (WNN) multimodal analyses [33]. Clustering was performed with the FindMultiModalNeighbors (RNA dimensions = 19, ADT dimensions = 12) and FindClusters (resolution = 0.4) functions. A mononuclear phagocyte subset was created. From the subsetted data, both RNA and ADT assays were rescaled and renormalized before constructing a new WNN graph with FindMultiModalNeighbors. Clustering was performed with the FindMultiModalNeighbors (RNA dimensions = 21, ADT dimensions = 12) and FindClusters (resolution = 0.4) functions.

### Bioinformatics: differential expression

Differential Expression was performed with the Wilcoxon Rank Sum test using the FindMarkers function within Seurat. RNA features were included in differential expression if they were expressed in at least 10% of cells and had log2FC > 0.59 (1.5-fold) between the cluster of interest and all other cells. The 1.5-fold cutoff was chosen so that were enough differentially expressed genes in each cluster for pathway analysis. Differential expression for ADT features was performed without cutoff. To identify biological pathways associated with different macrophage populations, differentially expressed genes from macrophage clusters were input into Ingenuity Pathway Analysis (QIAGEN Inc., https://digitalinsights.qiagen.com/IPA). Canonical pathways terms with a z-score of > 2, a *p*-value of < 0.01, and at least 4 differentially expressed genes in the pathway grouping were included in visualizations.

### Statistics

Data were checked for normality using the Shapiro–Wilk test and the appropriate parametric or non-parametric test was chosen for each dataset. All distributions were parametric except for laser CNV studies. Flow cytometry data were analyzed using Brown–Forsythe and Welch ANOVA followed by Dunnett’s T3 multiple comparisons test (classical monocytes) or two-way ANOVA followed by Sidak’s multiple comparisons test (non-classical monocytes, macrophages, microglia, and dendritic cells). Laser CNV was compared using the Kruskal–Wallis test followed by Dunn’s multiple comparisons test. Choroidal sprouting area was compared using Student’s unpaired *t* test. Hypergeometric enrichment analysis was performed with the phyper function in the R stats (v4.1.0) package. Enrichment was deemed significant at the α = 0.05 level with a Bonferroni correction for testing multiple hypotheses.

## Results

**Fig. 1:**
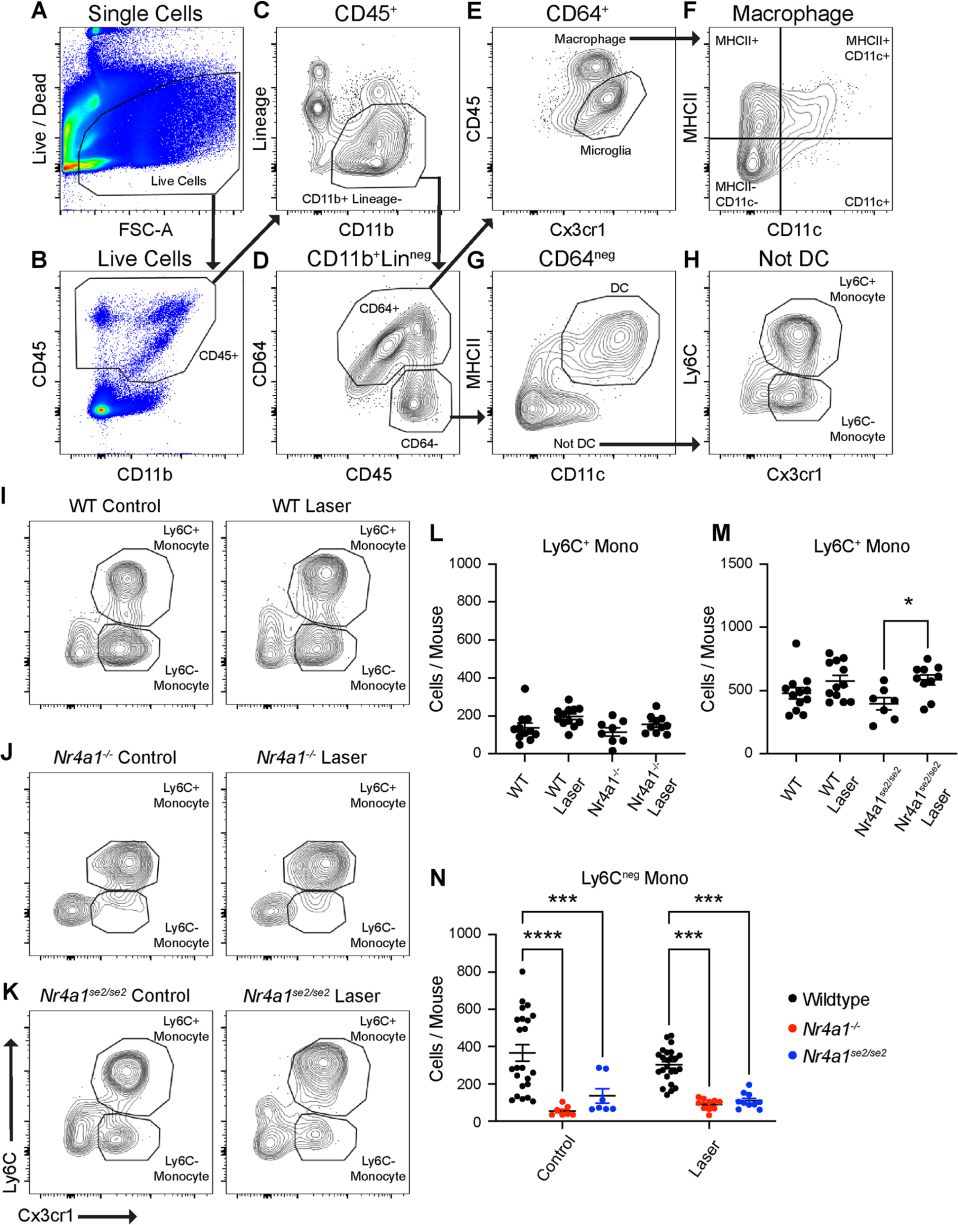
*Nr4a1*^*−/−*^ and *Nr4a1*^*se2/se2*^ mice are deficient in non-classical monocytes. **A–H** Gating strategy to include live cells (**A**), CD45^+^ cells (**B**), CD11b^+^Lin^neg^ cells (**C**), and CD64^+^ vs CD64^neg^ cells (**D**). Lineage (Lin) gate included T cells, B cells, NK cells, Eosinophils, and Neutrophils. **E** Gating of microglia vs macrophages from CD64^+^ cells. **F.** Gating of macrophage subtypes. **G** Gating of DCs from CD64^neg^ cells. **H** Gating of monocyte subtypes from non-DCs. **I–K** Representative monocyte plots from control and laser-treated WT (**I**) *Nr4a1*^*−/−*^ (**J**), and *Nr4a1*^*se2/se2*^ (**K**) eyes. **L, M** Number of classical monocytes from WT vs *Nr4a1*^*−/−*^ eyes (**L**) and WT and *Nr4a1*^*se2/se2*^ eyes (**M**). **N**: Numbers of non-classical monocytes from WT, *Nr4a1*^*−/−*^, and *Nr4a1*^*se2/se2*^ eyes. N = 7–12 mice per group. Data were analyzed using Brown–Forsythe and Welch ANOVA followed by Dunnett’s T3 multiple comparisons test (**L**, **M**) or two-way ANOVA followed by Sidak’s multiple comparisons test (**N**) *p < 0.05, **p < 0.01, ***p < 0.001, ****p < 0.0001. *DC* dendritic cell, *Mono* monocyte, *WT* wildtype

We performed multi-parameter flow cytometry on mouse retina and choroid–RPE–scleral complex to identify macrophage heterogeneity and confirm non-classical monocyte deficiency in *Nr4a1*^*−/−*^ and *Nr4a1*^*se2/se2*^ mice. Female mice were chosen for flow cytometry experiments because we previously showed that female mice have greater immune cell infiltration during laser-induced CNV compared to male mice, but the pattern of macrophage heterogeneity was unchanged [16]. Experiments were performed on Day 3 after laser, the peak of macrophage recruitment [16]. We first gated single, live cells (Fig. 1A). Next, CD45^+^ cells were identified (Fig. 1B). Mononuclear phagocytes were delineated as CD11b^+^ and Lin^neg^ (B220, CD4, CD8, NK1.1, Ly6G, SiglecF) cells (Fig. 1C). CD64 was used to isolate macrophages from non-macrophage cells (Fig. 1D). Microglia were defined as CD45^dim^Cx3cr1^high^ cells (Fig. 1E). Non-microglia were gated forward and divided into 4 subtypes by MHCII and CD11c expression (Fig. 1F). From CD64^neg^ non-macrophages, dendritic cells (DC) were identified as MHCII^+^CD11c^+^ cells (Fig. 1G). Non-DC were gated forward, classical and non-classical monocytes were defined as Cx3cr1^+^Ly6C^+^ and Cx3cr1^+^Ly6C^neg^ cells, respectively (Fig. 1H).

The above gating strategy was used on control and laser-treated wildtype (WT), *Nr4a1*^*−/−*^, and *Nr4a1*^*se2/se2*^ eyes. Representative terminal gates are shown in Fig. 1I–K. In WT and *Nr4a1*^*−/−*^ eyes, classical monocytes were unchanged (Fig. 1L). Classical monocytes were slightly increased in *Nr4a1*^*se2/se2*^ laser-treated eyes compared to control *Nr4a1*^*se2/se2*^ eyes (*p* < 0.05, Fig. 1M). Non-classical monocytes, however, were reduced by 84.9% (p < 0.0001) in *Nr4a1*^*−/−*^ control and 70.0% (*p* < 0.001) in *Nr4a1*^*−/−*^ laser-treated eyes (Fig. 1N). Non-classical monocytes were similarly reduced by 62.8% (*p* < 0.001) in *Nr4a1*^*se2/se2*^ control and 63.4% (*p* < 0.0001) in *Nr4a1*^*se2/se2*^ laser-treated eyes (Fig. 1N). No difference in non-classical monocyte numbers was detected between *Nr4a1*^*−/−*^ and *Nr4a1*^*se2/se2*^ eyes in control or laser-treated eyes. These data demonstrate that both *Nr4a1*^*−/−*^ and *Nr4a1*^*se2/se2*^ models are deficient in non-classical monocytes, as expected.

**Fig. 2:**
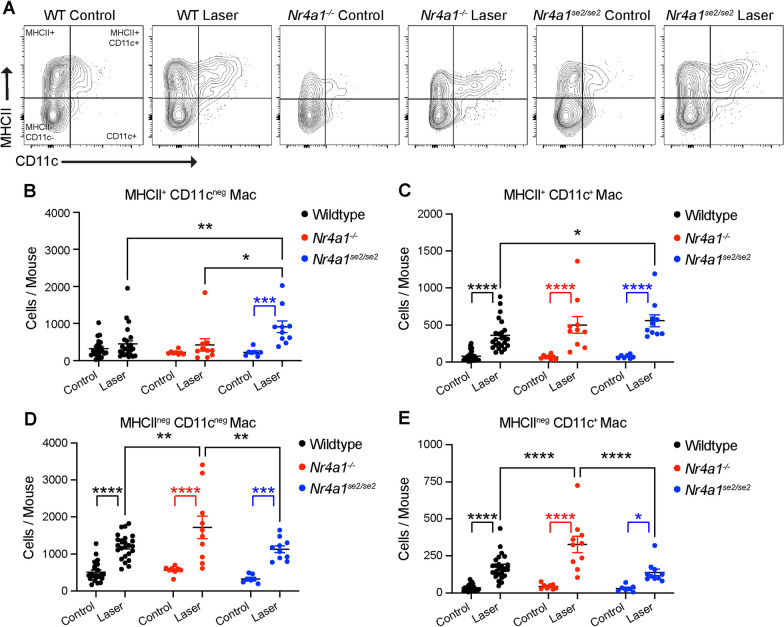
Macrophage subtypes are altered in *Nr4a1*^*−/−*^ and *Nr4a1*^*se2/se2*^ eyes. **A** Representative macrophage plots from control and laser-treated WT, *Nr4a1*^*−/−*^, and *Nr4a1*^*se2/se2*^ eyes. **B–E** Number of MHCII^+^CD11c^neg^ (**B**), MHCII^+^CD11c^+^ (**C**), MHCII^neg^CD11c^neg^ (**D**), and MHCII^neg^CD11c^+^ (**E**) macrophages from each group. N = 7–23 mice per group. Two-way ANOVA followed by Sidak’s multiple comparisons test *p < 0.05, **p < 0.01, ***p < 0.001, ****p < 0.0001. *Mac* macrophage, *WT* wildtype

Next, we compared macrophage subtypes between WT, *Nr4a1*^*−/−*^, and *Nr4a1*^*se2/se2*^ eyes to identify non-classical monocyte-derived macrophages (MDMs). Representative terminal gates are shown in Fig. 2A. MHCII^+^CD11c^neg^ macrophages were increased by laser treatment in *Nr4a1*^*se2/se2*^ eyes (*p* < 0.001), and this increase was greater than WT laser-treated (*p* < 0.01) and *Nr4a1*^*−/−*^ laser-treated eyes (*p* < 0.05, Fig. 2B). MHCII^+^CD11c^+^ macrophage counts were elevated by laser in WT, *Nr4a1*^*−/−*^, and *Nr4a1*^*se2/se2*^ eyes by 4.3-, 7.4-, and 7.5-fold, respectively (*p* < 0.0001 for all, Fig. 2C). MHCII^+^CD11c^+^ macrophages from *Nr4a1*^*se2/se2*^ laser-treated eyes were significantly greater than WT laser-treated eyes (*p* < 0.05, Fig. 2C). MHCII^neg^CD11c^neg^ macrophage numbers enlarged after laser in WT, *Nr4a1*^*−/−*^, and *Nr4a1*^*se2/se2*^ eyes by 2.4- (*p* < 0.0001), 3.0- (*p* < 0.0001), and 3.4-fold (*p* < 0.001), respectively (Fig. 2D). MHCII^neg^CD11c^neg^ macrophages from *Nr4a1*^*−/−*^ laser-treated eyes were significantly greater than WT laser-treated and *Nr4a1*^*se2/se2*^ laser-treated eyes (*p* < 0.01 for both, Fig. 2D). MHCII^neg^CD11c^+^ macrophages increased after laser in WT, *Nr4a1*^*−/−*^, and *Nr4a1*^*se2/se2*^ eyes by 5.0- (*p* < 0.0001), 7.4- (*p* < 0.0001), and 4.5-fold (*p* < 0.05), respectively (Fig. 2E). MHCII^neg^CD11c^+^ macrophages from *Nr4a1*^*−/−*^ laser-treated eyes were significantly greater than WT laser-treated and *Nr4a1*^*se2/se2*^ laser-treated eyes (*p* < 0.0001 for both, Fig. 2E). These data suggest that no macrophage subset was derived from non-classical MDMs. Additionally, *Nr4a1*^*se2/se2*^ laser-treated eyes have greater MHCII^+^CD11c^neg^ and MHCII^+^CD11c^+^ macrophages than WT laser-treated eyes, while *Nr4a1*^*−/−*^ laser-treated eyes showed more MHCII^neg^CD11c^neg^ and MHCII^neg^CD11c^+^ macrophages than WT laser-treated eyes.

**Fig. 3:**
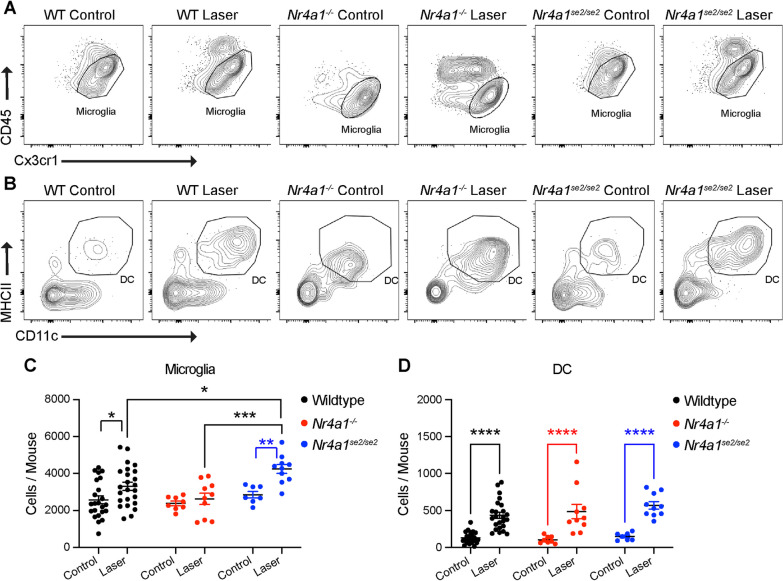
Numbers of microglia and DCs from *Nr4a1*^*−/−*^ and *Nr4a1*^*se2/se2*^ eyes. **A, B** Representative microglia (**A**) and DC (**B**) plots from control and laser-treated WT, *Nr4a1*^*−/−*^, and *Nr4a1*^*se2/se2*^ eyes. **C, D** Number of microglia (**C**) and DC (**D**) from each group. *N* = 7–23 mice per group. Two-way ANOVA followed by Sidak’s multiple comparisons test *p < 0.05, **p < 0.01, ***p < 0.001, ****p < 0.0001. *DC* dendritic cell, *WT* wildtype

Microglia and DC were compared between WT, *Nr4a1*^*−/−*^, and *Nr4a1*^*se2/se2*^ eyes. Representative terminal gates are shown in Fig. 3A, B for microglia and DC, respectively. Microglia numbers increased after laser by 1.3- (*p* < 0.05) and 1.5-fold (*p* < 0.01) in WT and *Nr4a1*^*se2/se2*^ eyes (Fig. 3C). *Nr4a1*^*se2/se2*^ laser-treated eyes demonstrated greater microglia than WT (*p* < 0.05) and *Nr4a1*^*−/−*^ laser-treated eyes (*p* < 0.001). DC numbers were elevated by 3.3-, 4.6-, 3.8-fold after laser treatment in WT, *Nr4a1*^*−/−*^, and *Nr4a1*^*se2/se2*^ eyes, respectively (*p* < 0.0001 for all, Fig. 3D), with no differences between genotypes. These data suggest that *Nr4a1*^*se2/se2*^ laser-treated eyes have slightly greater microglia, while DCs were equally increased after laser in all genotypes.

**Fig. 4:**
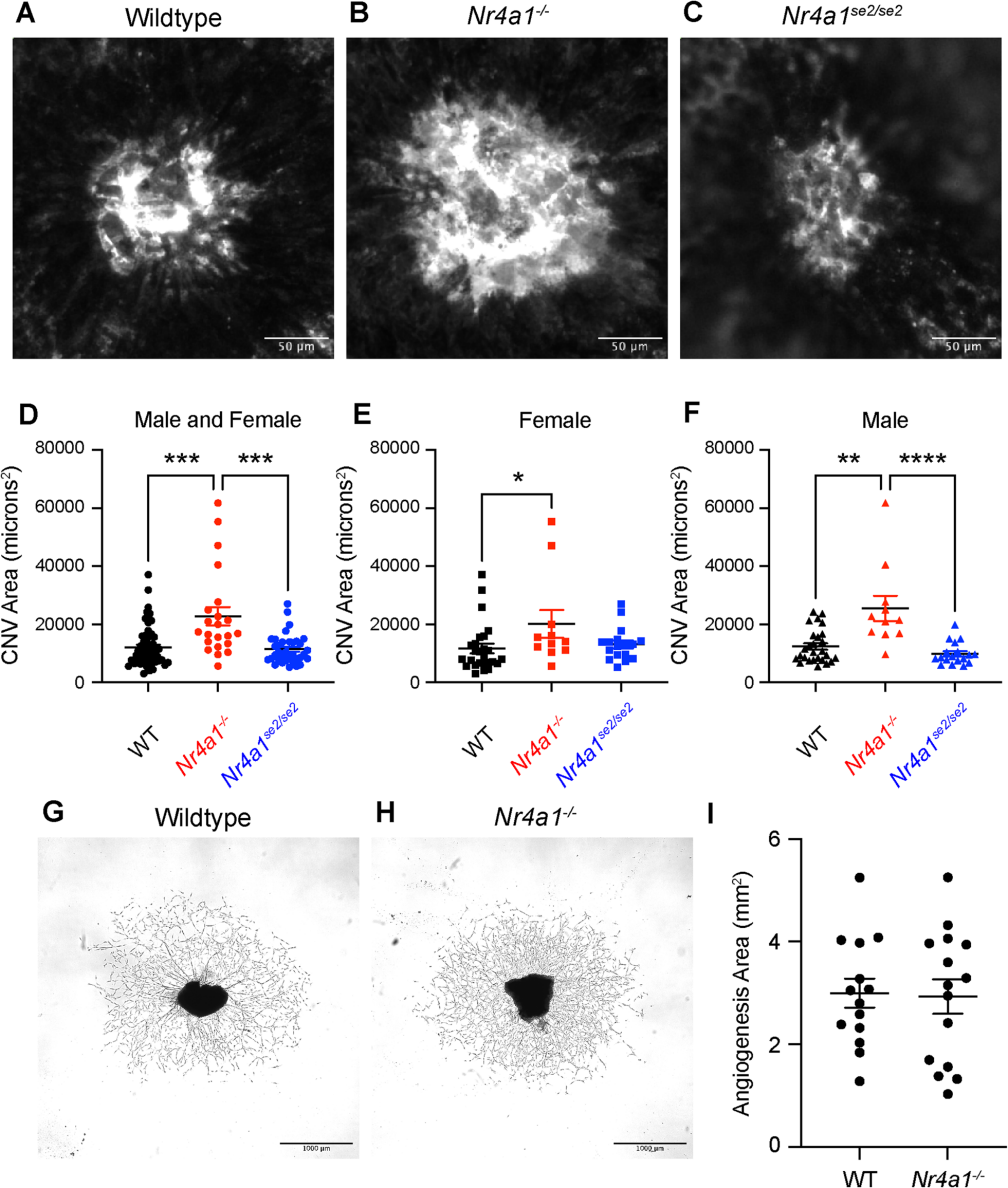
*Nr4a1*^*−/−*^ mice show increased laser-induced CNV area. **A–C** Representative choroidal wholemounts of a single CNV lesion from WT, *Nr4a1*^*−/−*^, and *Nr4a1*^*se2/se2*^ mice. **D–F** Laser-induced CNV area per mouse for all animals, female, and male mice. *N* = 22–53 mice per group. Kruskal–Wallis test followed by Dunn’s multiple comparisons test *p < 0.05, **p < 0.01, ***p < 0.001, ****p < 0.0001. **G, H** Representative choroidal sprouts from WT and *Nr4a1*^*−/−*^ eyes. **I** Average angiogenesis area was equivalent between groups. *N* = 14–15. Student’s unpaired t test

We next measured laser-induced CNV area in WT, *Nr4a1*^*−/−*^, and *Nr4a1*^*se2/se2*^ mice. Representative choroidal wholemounts stained for ICAM2 are shown in Fig. 4A–C on Day 7 after laser injury. We found that *Nr4a1*^*−/−*^ mice showed 1.9- and 2.0-fold greater CNV area compared to WT (*p* < 0.001) and *Nr4a1*^*se2/se2*^ mice (*p* < 0.001), respectively (Fig. 4D). *Nr4a1*^*se2/se2*^ mice showed no change in CNV area compared to WT mice. This effect was not sex-specific as *Nr4a1*^*−/−*^ mice demonstrated 1.7- (*p* < 0.05) and 2.1-fold (*p* < 0.01) greater CNV area compared to WT mice in female (Fig. 4E) and male (Fig. 4F) mice, respectively. These data suggest that non-classical monocytes and non-classical MDMs play no significant role in the laser-induced CNV model. Additionally, these data suggest that MHCII^neg^CD11c^neg^ and MHCII^neg^CD11c^+^ macrophages, which were increased in *Nr4a1*^*−/−*^ laser-treated eyes, may promote the laser-induced CNV model.

Since *Nr4a1*^*−/−*^ is also expressed in choroidal endothelial cells [34, 35], we performed the choroidal sprouting assay with tissue from both WT and *Nr4a1*^*−/−*^ mice. The choroidal sprouting assay tests endothelial cell growth potential by measuring spontaneous sprouting angiogenesis of choroid–RPE–scleral pieces in Matrigel [36]. We found that WT and *Nr4a1*^*−/−*^ mice displayed equal choroidal sprouting angiogenesis (Fig. 4G–I), suggesting that endothelial cells from both genotypes have equal capacity for migration, proliferation, and tube formation.

**Fig. 5:**
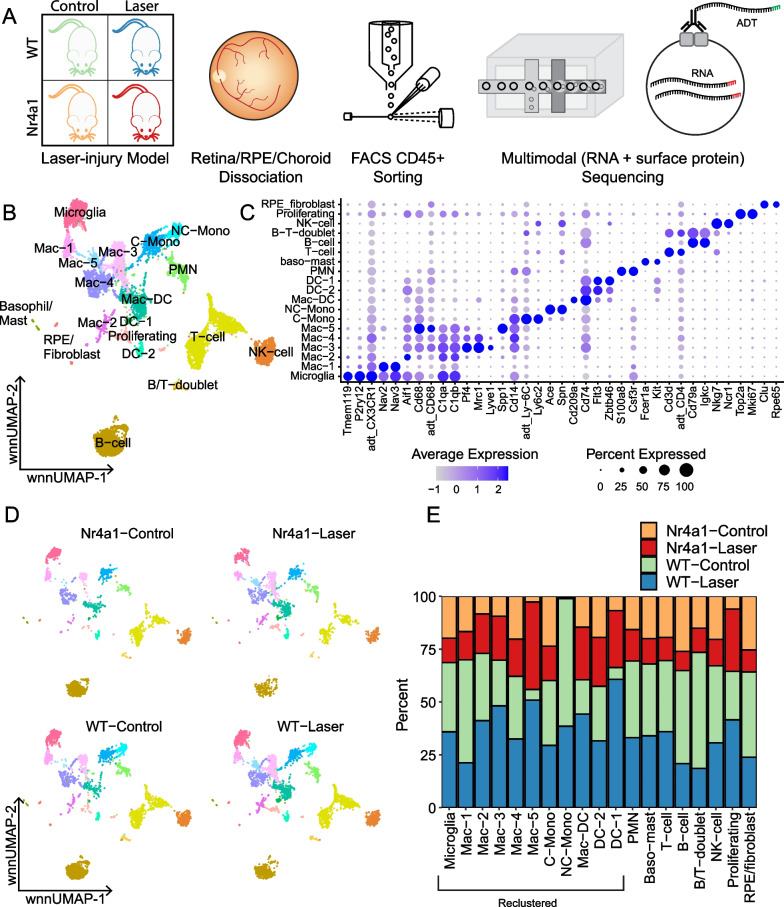
Multimodal sequencing of CD45^+^ immune cells. **A** Experimental overview. Retina/RPE/choroid/sclera was isolated from the four experimental groups before CD45^+^ FACS sorting and subsequent multimodal (RNA + surface protein) sequencing. **B** Weighted nearest neighbor (WNN) integration of RNA and ADT assays was performed before dimensionality reduction with uniform manifold approximation and projection (UMAP). **C** Canonical marker genes are displayed for each cellular cluster in a dot plot. **D** The contribution of the experimental conditions to each cluster is visualized in the faceted wnnUMAP plot. **E** The contribution of the four experimental groups to each cluster is visualized with a stacked bar chart. *Mac* macrophage, *C-mono* classical monocyte, *NC-mono* non-classical monocyte, *DC* dendritic cell, *PMN* polymorphonuclear neutrophil, *NK-cell* natural killer cell

Next, we performed multimodal single-cell RNA-sequencing (scRNA-seq) and Cellular Indexing of Transcriptomes and Epitopes sequencing (scCITE-seq) to analyze simultaneous RNA and protein expression from control and laser-treated WT and *Nr4a1*^*−/−*^ mice. We set out to determine if the increased MHCII^neg^CD11c^neg^ and/or MHCII^neg^CD11c^+^ macrophages from *Nr4a1*^*−/−*^ eyes observed from flow cytometry are pro-angiogenic and may lead to greater CNV area. We harvested eyes from 22 female WT and *Nr4a1*^*−/−*^ control mice, and 11–12 wildtype and *Nr4a1*^*−/−*^ laser-treated mice on Day 3 after laser injury. Female mice were chosen to align our findings with our multi-parameter flow cytometry studies. Eyes were pooled from each group to yield a biologically diverse sample. Eyes were dissected to isolate retina and RPE/choroid/sclera complex, digested into single cell suspension, and live, CD45^+^ cells were isolated by fluorescence-activated cell sorting (FACS) for scRNA-seq and scCITE-seq (Fig. 5A). After mapping reads to the genome and filtering low-quality cells, a total of 11,624 cells were recovered for downstream analysis (*n* = 4008 WT-laser, *n* = 1675 *Nr4a1*-laser, *n* = 3863 WT-control, *n* = 2078 *Nr4a1*-control). Next, we integrated the RNA and antibody derived tag (ADT; subsequently referred to as proteomic) data and performed clustering and dimensionality reduction (Fig. 5B). Clusters were classified into cell types based on the expression of previously described marker genes [18] and surface proteins (Additional files 3, 4: Table S1, S2), and all major immune cell populations were identified (Fig. 5C). We compared the contributions of the four experimental groups to each cluster (Fig. 5D, E). As expected, the WT control and WT laser groups had greater contributions to the non-classical monocyte cluster (NC-Mono, cyan), with almost no cells coming from either *Nr4a1*^*−/−*^ group. Similarly, the WT and *Nr4a1*^*−/−*^ laser groups contributed almost all the cells observed in the *Spp1*^+^ Mac-5 cluster (light blue), with only rare cells from the control groups.

**Fig. 6:**
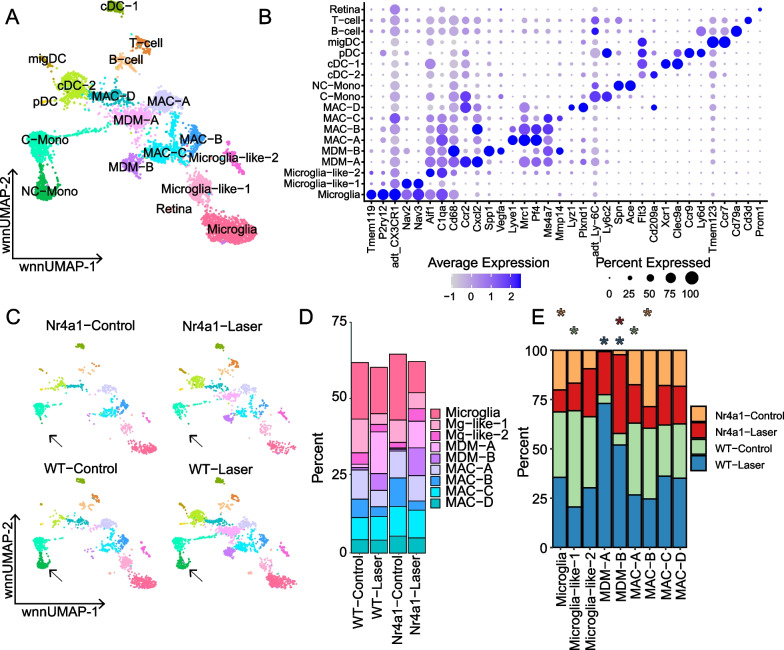
Multimodal sequencing of reclustered mononuclear phagocytes. **A** Reclustered mononuclear phagocytes on wnnUMAP. **B** Canonical marker genes are displayed for each cellular cluster in a dot plot. **C** The contribution of the experimental conditions to each cluster is visualized in the faceted wnnUMAP plot. The black arrow indicates the absence of the NC-mono cluster (green) from *Nr4a1*^*−/−*^ groups. **D** The distribution of cells within each experimental group is visualized with a stacked bar chart, including only macrophage and microglia clusters. **E** The contribution of the four experimental groups to each cluster is visualized with a stacked bar chart. Hypergeometric distribution analysis was performed to quantify if an experimental condition was significantly enriched in each cluster (asterisks). Asterisks are colored according to experimental condition and placed above a cluster if the enrichment test was significant at the α = 0.05 level after Bonferroni correction. *Mac* macrophage, *MDM* monocyte-derived macrophage, *C-mono* classical monocyte, *NC-mono* non-classical monocyte, *cDC* classical dendritic cell, *pDC* plasmacytoid dendritic cell, *migDC *migratory dendritic cell

To study macrophage subpopulations during the laser CNV response in more detail, we rescaled, reclustered, and performed a new dimensionality reduction on the mononuclear phagocyte (microglial, monocyte, macrophage, and dendritic cell) population (Fig. 6A). A total of 5586 cells were included in this reclustered object (*n* = 2124 WT-laser, *n* = 993 *Nr4a1*-laser, *n* = 1625 WT-control, *n* = 844 *Nr4a1*-control). Cells were again classified into clusters based on previously described gene expression patterns (Additional files 5, 6: Tables S3, S4). We identified three clusters of microglia or microglia-like cells, all of which demonstrated protein expression of CX3CR1 and RNA expression of a combination of *Tmem119, P2ry12, Nav2, Nav3,* and/or *C1qa* (Fig. 6B)*.* We identified two clusters of monocyte-derived macrophages (MDM); MDM-A expressed high levels of *Ccr2* and *Cxcl2*, while MDM-B demonstrated high expression of *Vegfa* and *Spp1*. There were four *Cd68*^+^*Aif1*^+^ macrophage clusters (MAC A-D), a *Ccr2*^+^*Ly6c2*^+^Ly6C^+^ classical monocyte cluster (C-Mono), a *Spn*^+^*Ace*^+^ non-classical monocyte cluster (NC-Mono), and four *Flt3*^+^ clusters of dendritic cells (DCs).

Next, we determined the contribution of the four experimental groups to each cluster (Fig. 6C–E). Like the original clustering, *Nr4a1*^*−/−*^ mice in the reclustered object contributed only rare cells to the non-classical monocyte cluster (Fig. 6C, green). Similarly, both the WT and *Nr4a1*^*−/−*^ laser groups contributed almost all the cells to the MDM-A (*Ccr2*^+^*Cxcl2*^+^) and MDM-B (*Vegfa*^+^*Spp1*^+^) clusters, justifying their nomenclature as MDMs. MDM-B consisted of 5.5% and 9.1% of all cells recovered from the WT and *Nr4a1*^*−/−*^ laser-treated groups, respectively (Fig. 6D). To formally compare the proportion of cells in each cluster across the four experimental conditions, we performed a hypergeometric distribution analysis (Fig. 6E). MDM-A was significantly enriched in the WT laser condition while MDM-B (*Vegfa*^+^*Spp1*^+^) was significantly enriched in both WT laser and *Nr4a1*^*−/−*^ laser conditions. These data suggest that MDM-B made up a larger percentage of total macrophages from *Nr4a1*^*−/−*^ laser-treated mice and may be contributing to angiogenesis and larger CNV size.

**Fig. 7:**
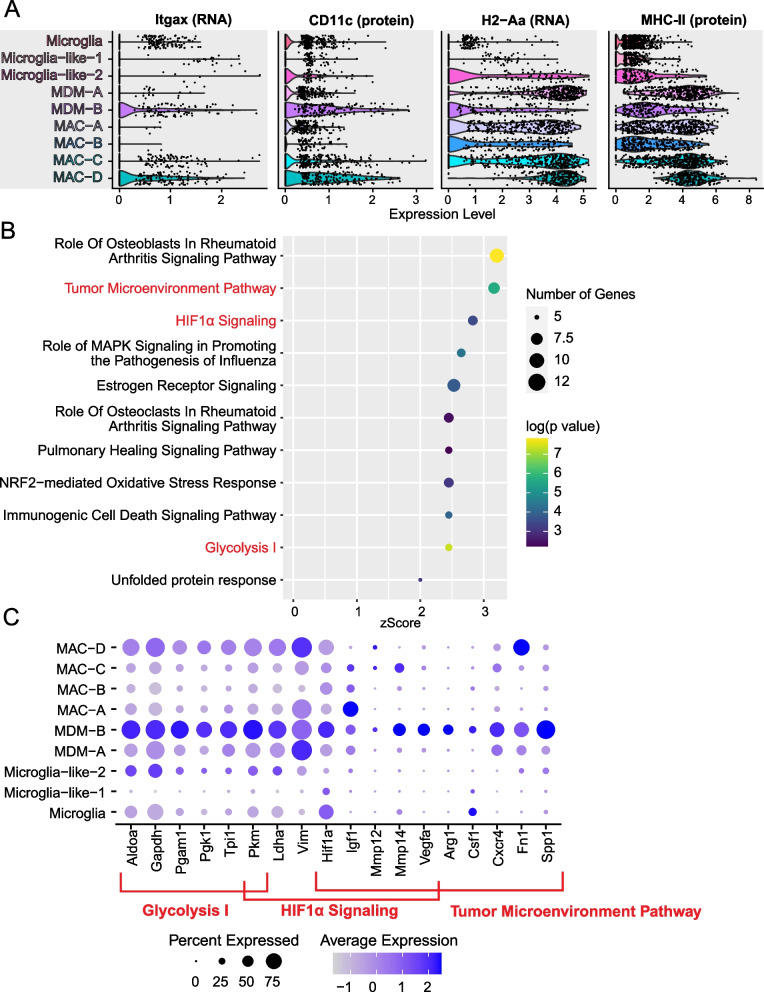
Canonical pathway enrichment of the pro-angiogenic MDM-B cluster. **A** Expression of Itgax/CD11c and H2-A1/MHCII in macrophage clusters. **B** Canonical pathway terms enriched in the MBM-B cluster. **C** Dot plot of MDM-B enriched genes in the glycolysis I, tumor microenvironment, and HIF1α signaling pathways

As cluster MDM-B expressed *Vegfa* and was enriched in both laser conditions, we next assessed the expression of MHCII (*H2-Eb1*) and CD11c (*Itgax*) to determine if MDM-B corresponded to MHCII^neg^CD11c^neg^ and/or MHCII^neg^CD11c^+^ macrophages, which were greater in *Nr4a1*^*−/−*^ laser-treated eyes compared to WT laser-treated eyes by multi-parameter flow cytometry (Fig. 2). Of all the macrophage populations, MDM-B showed high levels of *Itgax* and CD11c expression and some of the lowest *H2-Eb1* and MHCII expression levels, suggesting that MDM-B is likely included within the MHCII^neg^CD11c^+^ macrophage population (Fig. 7A).

To investigate if MDM-B is pro-angiogenic, we performed differential expression to identify genes enriched in the MDM-B cluster and all other macrophage populations. Genes with an absolute enrichment greater than 1.5-fold were input into Ingenuity Pathway Analysis. Enriched canonical pathway terms that support the hypothesis that the MDM-B cluster is pro-angiogenic include glycolysis I (*z*-score of 2.45), tumor microenvironment (*z*-score of 3.16), and hypoxia inducible factor 1α (HIF1α, *z*-score of 2.83) signaling pathways (Fig. 7B). Canonical pathway enrichment for other macrophage clusters can be found in Additional file 1: Fig. S1.

A pro-glycolytic expression profile has been previously associated with macrophages in the laser-induced CNV and oxygen-induced retinopathy models [37–39]. MDM-B macrophages demonstrated increased expression of 6 glycolysis genes (Fig. 7C), including the aldolase gene *Aldoa*, which has been shown to regulate the VEGF signaling pathway [40]. Pathway enrichment for the tumor microenvironment suggests that MDM-B expressed a similar transcriptional profile to tumor-associated macrophages, which have been shown to promote angiogenesis [41]. Tumor microenvironment genes linked to ocular angiogenesis include *Spp1, Csf1*, and *Cxcr4* (Fig. 7C). In AMD patients with the 10q26 risk allele, SPP1 (secreted phosphoprotein 1) expression is greater in macrophages, and SPP1^+^ Macs are detectable in human CNV membranes [42, 43]. Furthermore, *Spp1*^*−/−*^ mice demonstrate reduced CNV size [42]. Inhibition of CSF1 (colony stimulating factor 1) results in both choroidal vascular atrophy at steady state and smaller CNV area [44, 45]. Additionally, the CXCL12-CXCR4 (chemokine ligand-receptor) pathway stimulates ocular angiogenesis in multiple models, including laser-induced CNV [46, 47]. Finally, HIF1a is a transcriptional activator that increases expression of pro-angiogenic targets, including glycolysis genes and *Vegfa* in response to ischemia (Fig. 7C). In addition to *Hif1a* and *Vegfa* upregulation, MDM-B showed differential expression of *Mmp12*, *Mmp14*, and *Igf1*, which were also part of the tumor microenvironment pathway (Fig. 7C). The matrix metalloproteinase genes *Mmp12* and *Mmp14* have been shown to be pro-fibrotic and pro-angiogenic in the eye, respectively [48, 49]. IGF-1 (insulin-like growth factor 1) levels increase during laser-induced CNV and IGF-1 augments CXCL12- and VEGFA-induced angiogenesis [50]. In addition, *Igf1* inhibition suppresses microglia-driven aortic and choroidal sprouting angiogenesis [39].

We next investigated genes that were previously differentially expressed in hyper-activated *Nr4a1*^*−/−*^ macrophages, which included decreased *Arg1* levels and increased expression of *Tnf*, *Il12*, *Nos2*, and *Cd36* [27]. We found that MDM-B demonstrated significantly more *Arg1* and less *Tnf* expression with no changes in *Il12b*, Nox2, or *Cd36* (Additional file 2: Fig S2). Similarly, when comparing *Nr4a1*^*−/−*^ laser-treated sample to WT laser-treated sample within the MDM-B cluster, none of the above genes were differentially expressed. Collectively, these gene expression profiles suggest that the MDM-B cluster, which is enriched from *Nr4a1*-lasered mice, expresses a pro-angiogenic transcriptional profile which is unique compared to macrophage hyper-activation in *Nr4a1*^*−/−*^ mice.

## Discussion

In this report, we investigated the role of non-classical monocytes and NR4A1 in the laser-induced CNV model of neovascular AMD. We first confirmed that both *Nr4a1*^*−/−*^ and *Nr4a1*^*se2/se2*^ eyes were deficient in non-classical monocytes (Fig. 1). Next, we found that no macrophage subtype was depleted in either *Nr4a1*^*−/−*^ or *Nr4a1*^*se2/se2*^ eyes, suggesting that no macrophages are derived from non-classical monocytes during the laser-induced CNV model. Instead, *Nr4a1*^*se2/se2*^ eyes showed increased MHCII^+^CD11c^neg^ macrophage subtypes, while *Nr4a1*^*−/−*^ eyes demonstrated greater MHCII^neg^CD11c^+^ and MHCII^neg^CD11c^neg^ macrophage numbers (Fig. 2). Importantly, *Nr4a1*^*−/−*^ mice displayed increased CNV area compared to WT and *Nr4a1*^*se2/se2*^ mice (Fig. 4), suggesting that MHCII^neg^CD11c^+^ and/or MHCII^neg^CD11c^neg^ macrophages may be pro-angiogenic. Single-cell RNA-seq and CITE-seq identified cluster MDM-B that was enriched from *Nr4a1*^*−/−*^ eyes and expressed *Itgax*/CD11c with low MHCII levels (Figs. 6, 7). Furthermore, MDM-B expressed a transcriptome that included enrichment for HIF1a signaling, glycolysis, and the tumor microenvironment, which is known to be pro-angiogenic. These data suggest that non-classical monocytes are dispensable in the laser-induced model, and that loss of NR4A1 shifts the transcriptomic profile of classical MDMs toward an angiogenesis-promoting phenotype.

Our data that MDM-B, which was CD11c^+^, is pro-angiogenic agree with several prior reports. In scRNA-seq from human RPE and choroid, we previously found that CD11C^+^ macrophages are enriched from patients with neovascular AMD and express high levels of *VEGFA* and *CXCR4* [16], two genes that were upregulated in MDM-B in this study. In addition, using scRNA-seq from the laser-induced CNV model, we previously identified that *Spp1*^+^ macrophages express *Itgax*/CD11c and a pro-angiogenic transcriptome—including *Spp1*, *Vegfa*, glycolysis genes, *Mmp12*, *Mmp14*, and *Cxcr4*—which were all shared with MDM-B in this study [18]. Furthermore, CD11c^+^ macrophage depletion decreases CNV area, demonstrating the pro-angiogenic role for CD11c^+^ macrophages [18]. These data from an independent scRNA-seq study on retina and choroid, as opposed to whole eye, confirm the rigor and reproducibility of pro-angiogenic CD11c^+^ macrophages in the laser-induced CNV model. Finally, this common pro-angiogenic transcriptome for macrophages is shared with the oxygen-induced retinopathy model. A prior report found that *Spp1*^+^ microglia, although no fate mapping was performed to confirm this identity, also express multiple glycolysis genes, *Hif1a*, and *Igf1*, in common with MDM-B [39]. These data suggest that CD11c^+^*Spp1*^+^ macrophages are potentially pro-angiogenic across species and diseases.

Loss of NR4A1 leads to a disparate transcriptional profile compared to previously reported macrophage hyper-activation in *Nr4a1*^*−/−*^ mice. In an atherosclerotic model, macrophages from *Nr4a1*^*−/−*^ mice show lower *Arg1* levels and greater expression of *Tnfa*, *Il12*, *Nos2*, and *Cd36* [27]. Alternatively, MDM-B demonstrated significantly greater *Arg1* and lower *Tnfa* expression with no differential expression of *Il12*, *Cd36*, or *Nos2*. Similarly, when comparing *Nr4a1*^*−/−*^ laser-treated sample to WT laser-treated sample within the MDM-B cluster, none of the canonical macrophage hyper-activation genes were differentially expressed. These suggest that the pro-angiogenic phenotype of *Nr4a1*^*−/−*^ macrophages is not due to macrophage hyper-activation, as previously reported.

Our data that *Nr4a1*^*se2/se2*^ mice showed no change in laser-induced CNV area suggest that non-classical monocytes play no significant role in the laser-induced CNV model. Therefore, the increased laser-induced CNV area found in *Cx3cr1*^*−/−*^ mice [21] is possibly related to microglia dysregulation. Microglia are known to migrate to the sub-retinal space to protect the RPE from injury [14], in support of the hypothesis that microglia may play homeostatic rather than pathologic roles in the laser-induced CNV model. Single-cell RNA-seq has now been performed 3 times, including this study, in the laser-induced CNV model. The first report sequenced immune cells from the retina, which primarily identified microglia [51]. One microglia cluster was enriched for wound healing in this prior study [51]. Similarly, our prior report found several microglia clusters, and one microglia cluster did demonstrate a pro-glycolytic transcriptional profile that has some similarities to *Spp1*^+^ macrophages [18]. In this study, microglia were enriched for tumor microenvironment, microglia-like-1 showed FGF signaling enrichment, and microglia-like-2 was pro-glycolytic but to a lesser degree than MDM-B (Additional file 1: Fig S1). Therefore, the role of microglia in the laser-induced CNV model remains open to debate, and definitive microglia-specific ablation studies are needed to understand the pathogenic vs protective potential roles for microglia in the sub-retinal space during laser-induced CNV.

This study has several limitations. We were only able to sequence 11,624 immune cells and 5,586 macrophages from posterior eye cups including retina, RPE, choroid, and sclera. This number is lower than our previously sequenced 34,215 cells from whole eyes despite using 22 control mice per each group and 11–12 laser-treated mice per group. It would have been ideal to sequence retina and RPE/choroid/sclera complex independently to delineate the contributions of each microenvironment. Moreover, greater cell numbers may have allowed us to identify additional heterogeneity within MDM-B or within the microglia clusters to gain further insights. Despite these limitations, we were able to reproducibly identify CD11c^+^ macrophages as pro-angiogenic. Second, we acknowledge that we did not deplete CD11c^+^ macrophages in this manuscript to directly test if CD11c^+^ macrophages promote CNV in *Nr4a1*^*−/−*^ mice. Third, although *Nr4a1*^*−/−*^ mice showed no change in choroidal sprouting angiogenesis compared to wildtype mice, the choroidal sprouting assay only tests the ability of endothelial cells to migrate, proliferate, and form tubes in Matrigel. Since we did not comprehensively characterize additional endothelial cell functions in *Nr4a1*^*−/−*^ mice during laser-induced CNV, loss of endothelial cell NR4A1 may still contribute to larger CNV lesions in *Nr4a1*^*−/−*^ mice. Fourth, since we did not perform myeloid-specific *Nr4a1* deletion, we cannot rule out a role for NR4A1 in other stromal cells, promoting CNV. Fifth, although NR4A1 activation could shift macrophage polarization toward a less angiogenic phenotype as a potential therapeutic target during neovascular AMD, this potential therapy would need to be highly specific for CNV-associated macrophages. NR4A1 activation outside of CNV lesions has the potential to reduce classical monocyte numbers, increase non-classical monocyte numbers, and potentially change the homeostatic function of tissue-resident macrophages. Instead, our data further implicate the pathogenic and pro-angiogenic function of CD11c^+^ macrophages during laser-induced CNV and potentially neovascular AMD.

## Conclusions

In summary, we demonstrated that no macrophage subtype is derived from non-classical monocytes and that non-classical monocytes play no significant role during laser-induced CNV. Additionally, NR4A1 deficiency alters the transcriptional profile of classical MDMs toward a multimodal pro-angiogenic profile including increased glycolysis gene expression, HIF1a-driven *Vegfa*, *Igf1*, *Mmp12*, and *Mmp14* expression, and upregulation of the tumor microenvironment pathway including *Spp1*, *Cxcr4*, and *Csf1*.

### Supplementary Information

**Additional file 1:** **Fig S1**. Ingenuity Pathway Analysis in all macrophage clusters. Canonical pathway terms for all macrophage and microglia clusters.

**Additional file 2:** **Fig S2**. Hyper-activation genes are not increased in MDM-B. Expression of macrophage hyper-activation genes in macrophage clusters.

**Additional file 3:** **Table S1**. Differential RNA expression for each cluster from all cells. Avg_log2FC = log2 fold change, pct.1 = percent of cells in cluster that express the gene, pc.2 = percent of all other cells that express the gene, *p*_val_adj = adjusted *p*-value for multiple comparisons, delta.pct = difference between pct.1 and pct.2. exp_”cluster” = average expression of the gene in the cluster, exp_world = average expression of the gene in all other cells.

**Additional file 4:** **Table S2**. Differential ADT expression for each cluster from all cells. Avg_log2FC = log2 fold change, pct.1 = percent of cells in cluster that express the gene, pc.2 = percent of all other cells that express the gene, *p*_val_adj = adjusted *p*-value for multiple comparisons, delta.pct = difference between pct.1 and pct.2. exp_”cluster” = average expression of the gene in the cluster, exp_world = average expression of the gene in all other cells.

**Additional file 5:** **Table S3**. Differential RNA expression for each cluster from mononuclear phagocytes. Avg_log2FC = log2 fold change, pct.1 = percent of cells in cluster that express the gene, pc.2 = percent of all other cells that express the gene, *p*_val_adj = adjusted *p*-value for multiple comparisons, delta.pct = difference between pct.1 and pct.2. exp_”cluster” = average expression of the gene in the cluster, exp_world = average expression of the gene in all other cells.

**Additional file 6:** **Table S4**. Differential ADT expression for each cluster from mononuclear phagocytes. Avg_log2FC = log2 fold change, pct.1 = percent of cells in cluster that express the gene, pc.2 = percent of all other cells that express the gene, *p*_val_adj = adjusted *p*-value for multiple comparisons, delta.pct = difference between pct.1 and pct.2, exp_”cluster” = average expression of the gene in the cluster, exp_world = average expression of the gene in all other cells.

**Additional file 7: Table S5**. Flow cytometry and immunofluorescence antibodies. Flow cytometry and immunofluorescence antibodies.

**Additional file 8:** **Table S6**. ADT antibodies. ADT antibodies.

## Data Availability

The datasets used and/or analyzed during the current study are available from the corresponding author on reasonable request.

## Abbreviations

AMD: Age-related macular degeneration
RPE: Retinal pigment epithelium
CNV: Choroidal neovascularization
VEGF: Vascular endothelial growth factor
MDM: Monocyte-derived macrophage
Lin: Lineage
DC: Dendritic cell
WT: Wildtype
scRNA-seq: Single-cell RNA-sequencing
scCITE-seq: Single-cell Cellular Indexing of Transcriptomes and Epitopes sequencing
FACS: Fluorescence-activated cell sorting
ADT: Antibody derived tag
NC-Mono: Non-classical monocyte
HIF: Hypoxia-inducible factor
SPP1: Secreted phosphoprotein 1
CSF1: Colony stimulating factor 1
IGF-1: Insulin-like growth factor 1
