# Supplementary figures and images for: NR4A1 deletion promotes pro-angiogenic polarization of macrophages derived from classical monocytes in a mouse model of neovascular age-related macular degeneration
Source: J Neuroinflammation. 2023 Oct 19;20:238. doi: 10.1186/s12974-023-02928-1 (PMC10588116; doi:10.1186/s12974-023-02928-1)

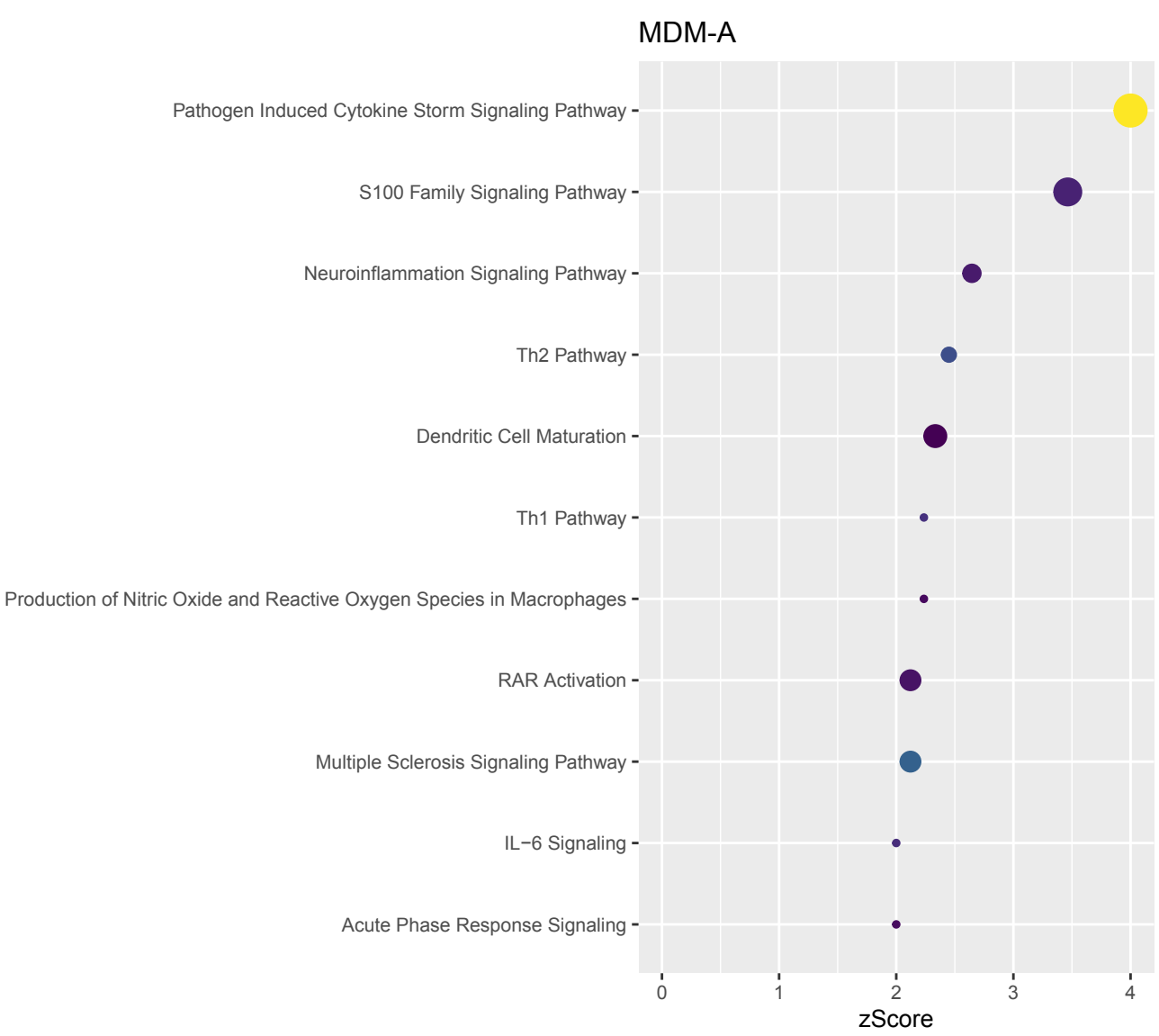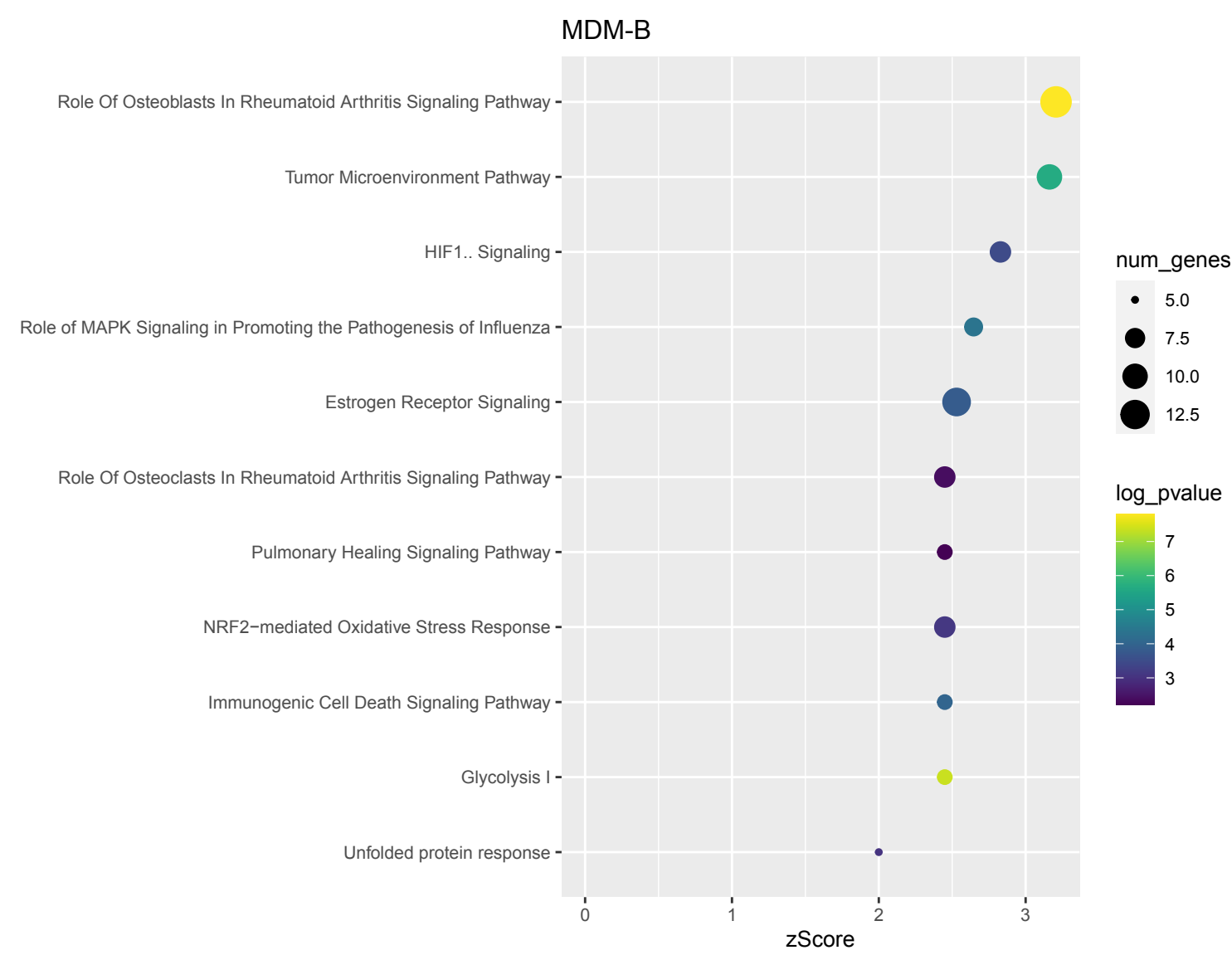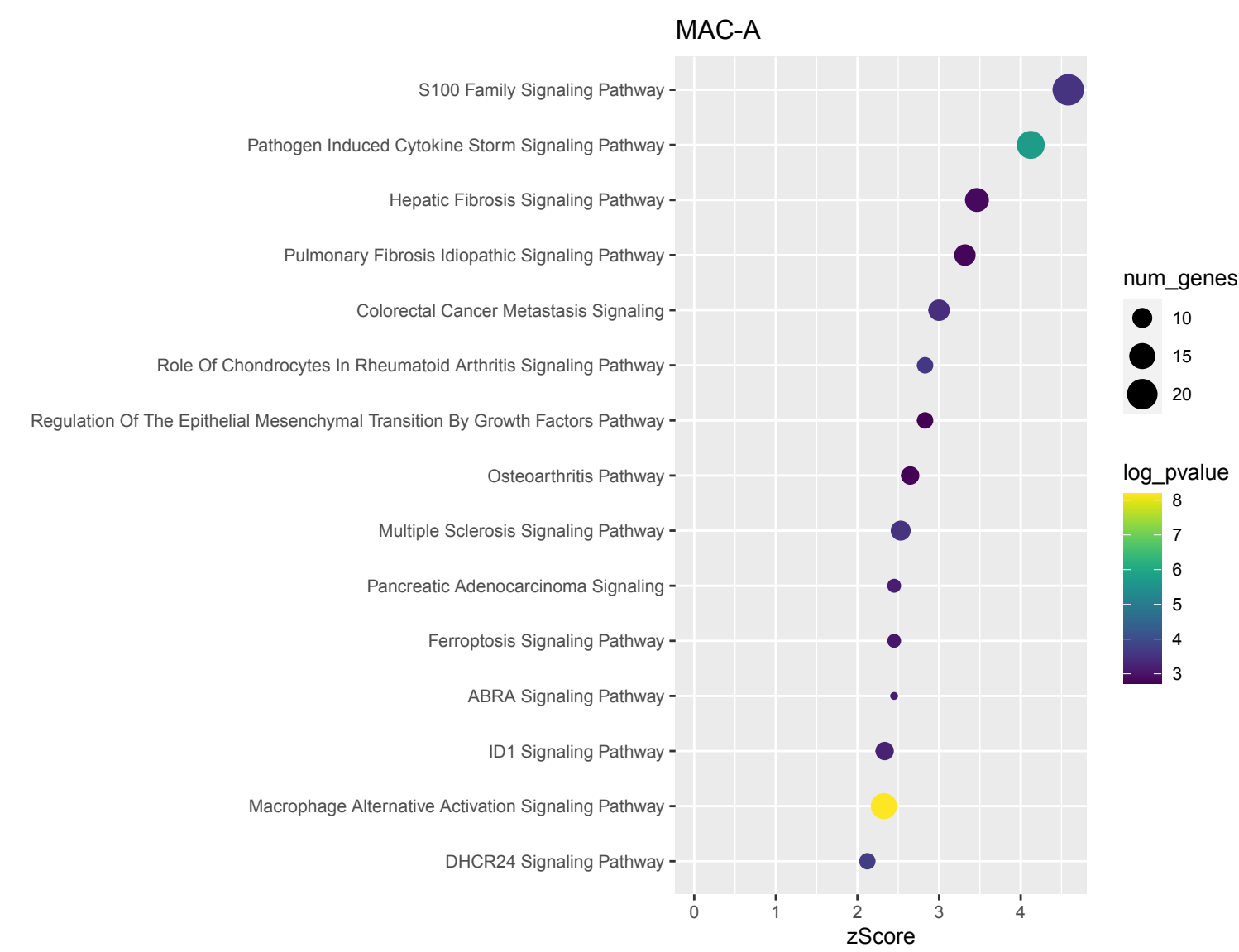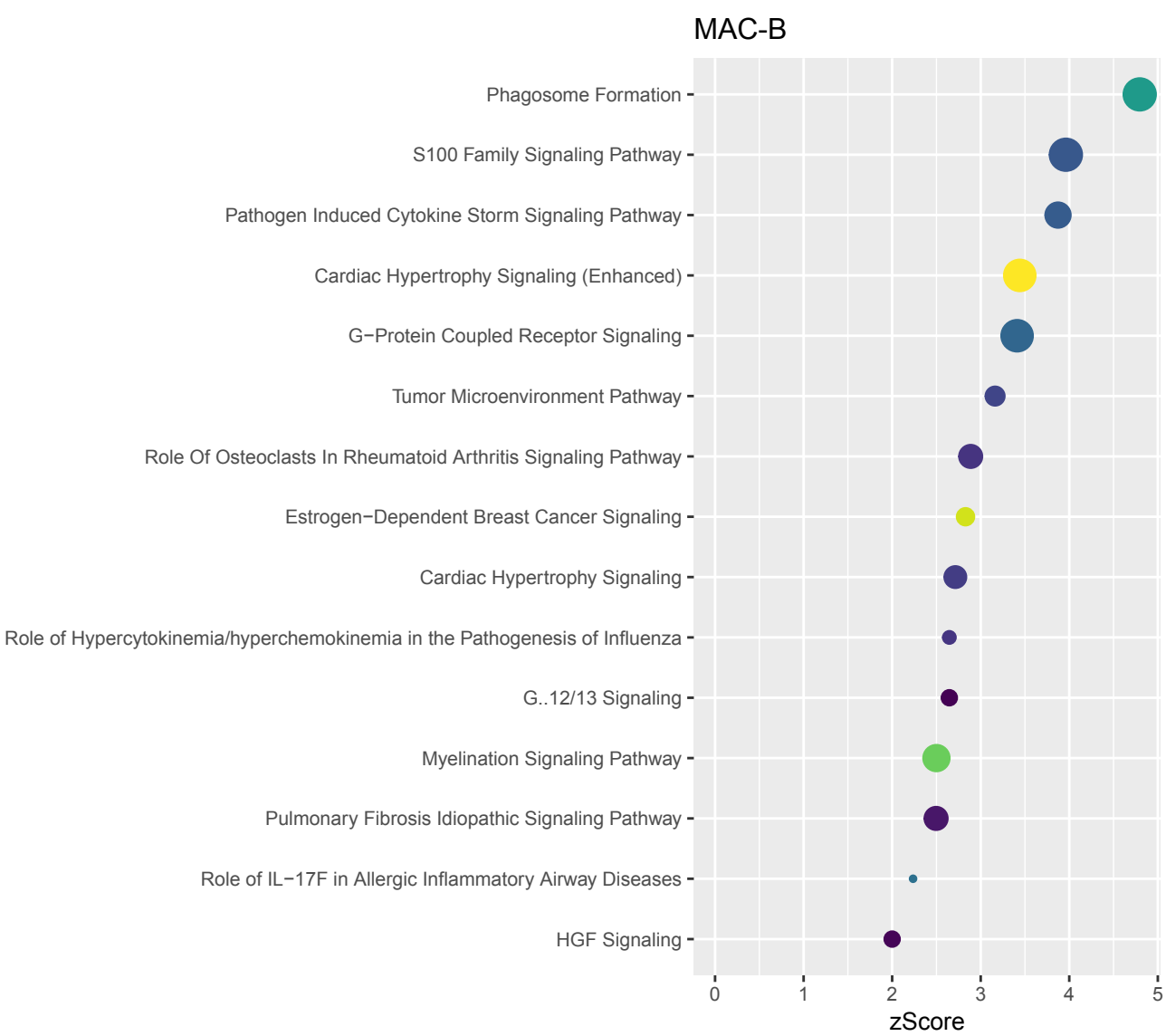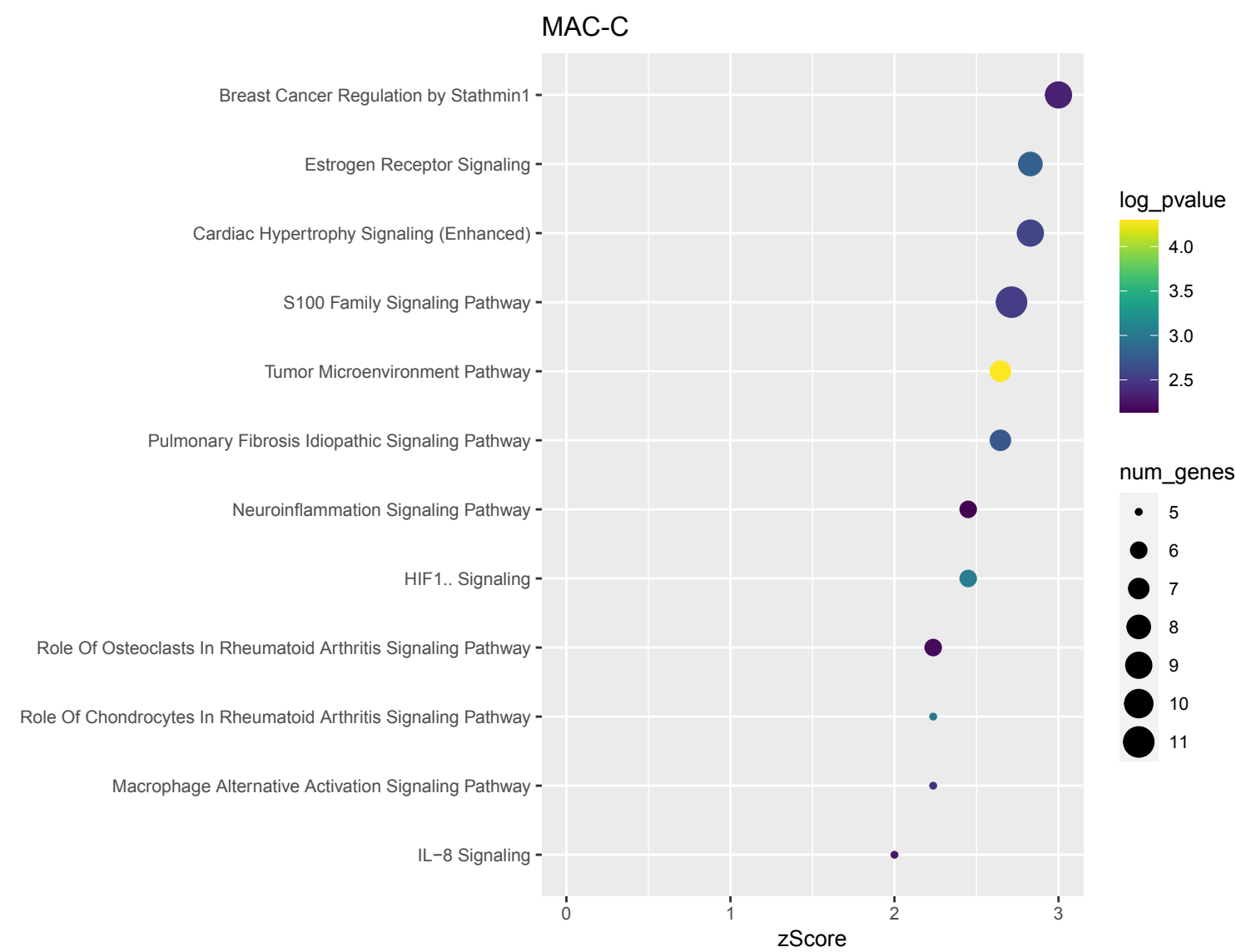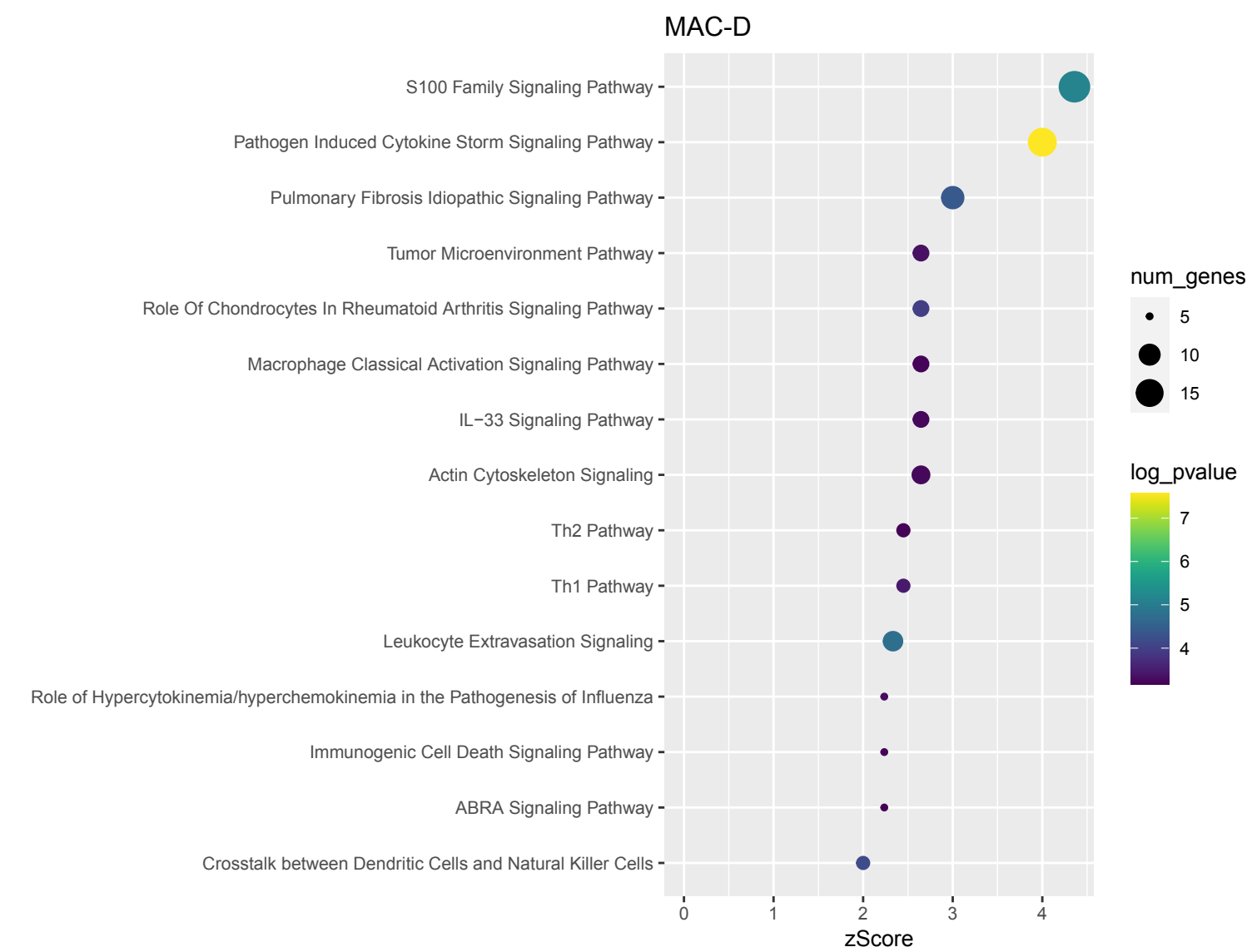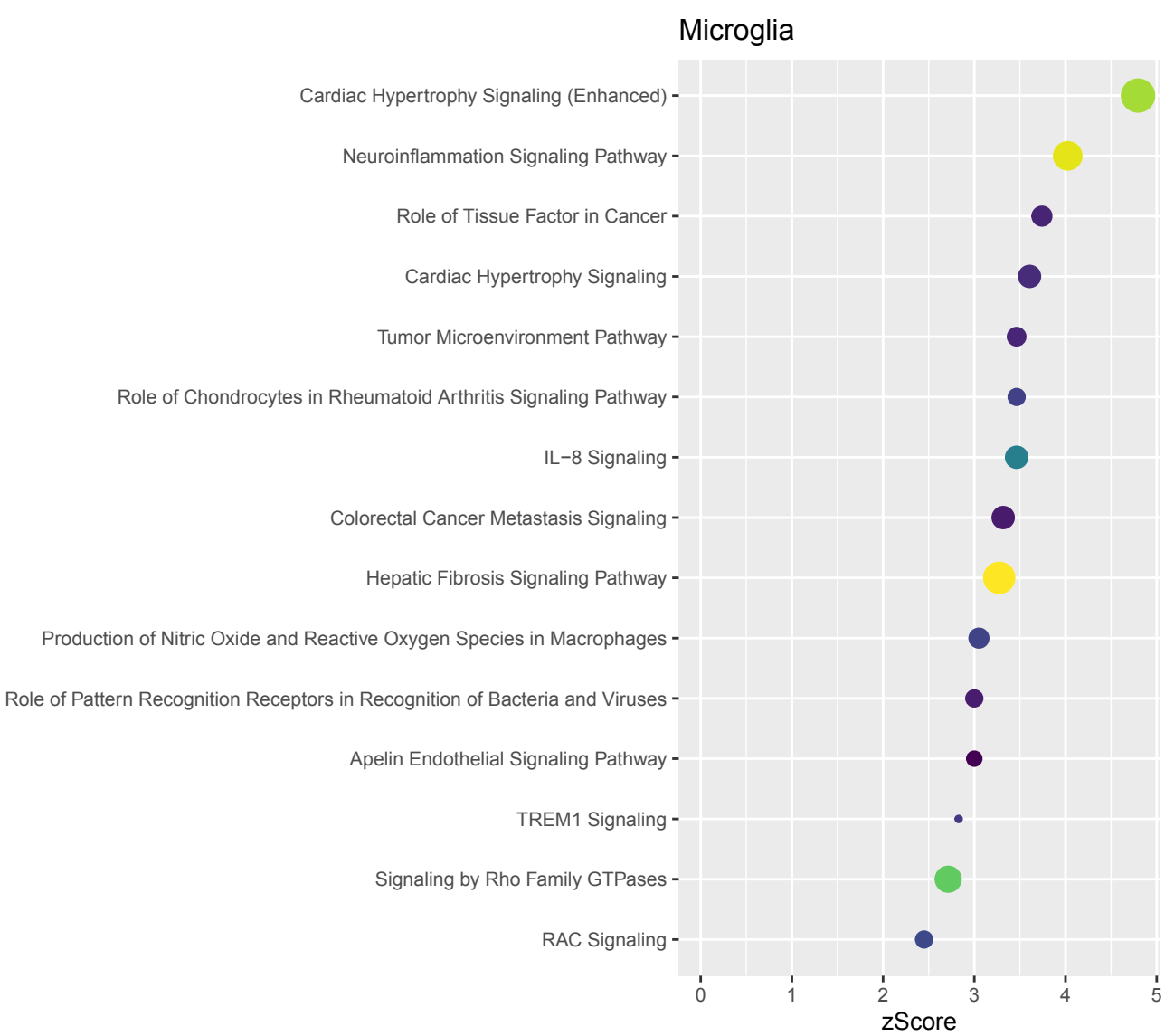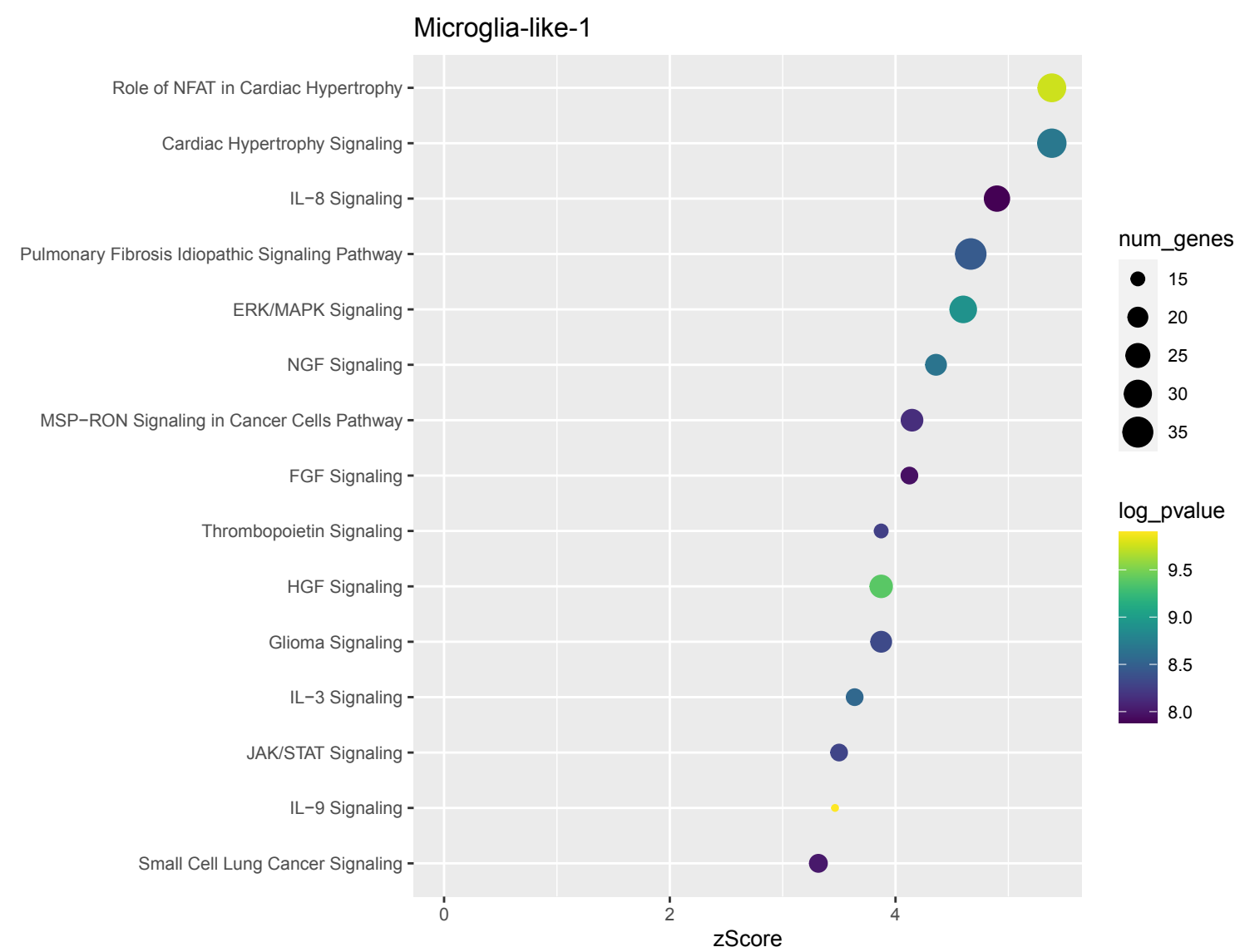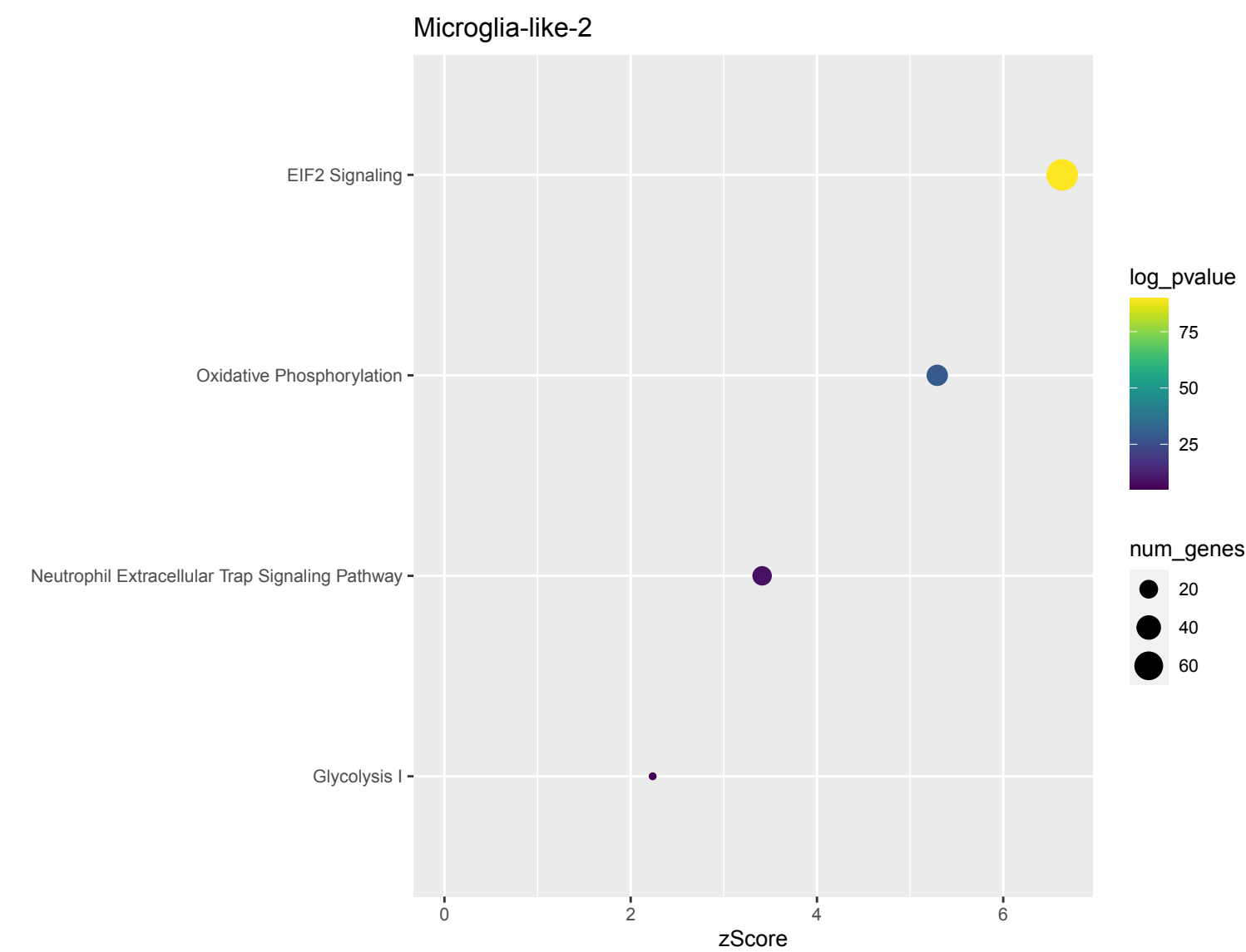

Supplement: Supplementary file 1 — Additional file 1: Fig S1. Ingenuity Pathway Analysis in all macrophage clusters. Canonical pathway terms for all macrophage and microglia clusters. [file 12974_2023_2928_MOESM1_ESM.pdf]

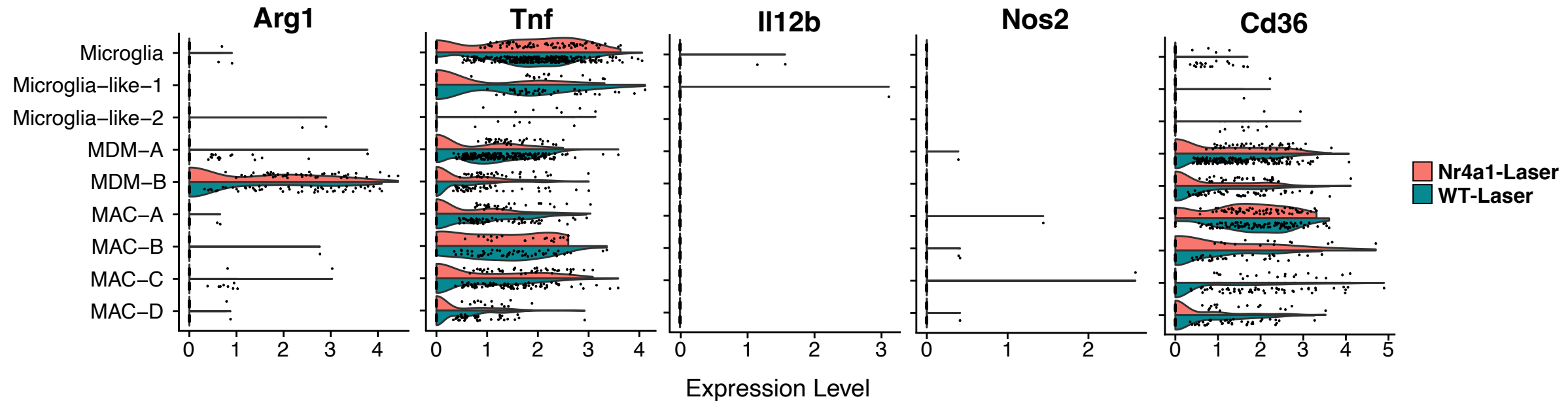

Supplement: Supplementary file 2 — Additional file 2: Fig S2. Hyper-activation genes are not increased in MDM-B. Expression of macrophage hyper-activation genes in macrophage clusters. [file 12974_2023_2928_MOESM2_ESM.pdf]
