# Supplementary material for: NR4A1 deletion promotes pro-angiogenic polarization of macrophages derived from classical monocytes in a mouse model of neovascular age-related macular degeneration
Source: J Neuroinflammation. 2023 Oct 19;20:238. doi: 10.1186/s12974-023-02928-1 (PMC10588116; doi:10.1186/s12974-023-02928-1)
Supplement: Supplementary file 7 — Additional file 7: Table S5. Flow cytometry and immunofluorescence antibodies. Flow cytometry and immunofluorescence antibodies. [file 12974_2023_2928_MOESM7_ESM.docx]

Table S5. Immunofluorescence and flow cytometry antibodies

| Target | Fluorophore | Manufacturer, Product Number, Dilution | Use |
| --- | --- | --- | --- |
| Fc block | - | BD Biosciences, 553142 | All Flow Cytometry |
| Aqua Live/Dead | AmCyan | ThermoFisher, 65-0866-14 | All Flow Cytometry |
| CD45 | Fitc | ThermoFisher**,**11-0451-82 | scRNA FACS |
| CD45 | BUV395 | BD Biosciences, 564279 | Flow Cytometry Analysis |
| CD64 | BV786 | BD Biosciences, 741024 | Flow Cytometry Analysis |
| CD11b | APC-Cy7 | BD Biosciences, 557657 | All Flow Cytometry |
| MHC II | AF700 | Biolegend, 107622 | Flow Cytometry Analysis |
| CD11c | BV421 | BD Biosciences, 562782 | Flow Cytometry Analysis |
| Cx3Cr1 | BV650 | Biolegend, 149033 | Flow Cytometry Analysis |
| CD4 | PE-CF594 | BD Biosciences, 562314 | Flow Cytometry Analysis |
| CD8 | PE-CF594 | BD Biosciences, 562315 | Flow Cytometry Analysis |
| SiglecF | PE-CF594 | BD Biosciences, 562757 | Flow Cytometry Analysis |
| B220 | PE-CF594 | BD Biosciences, 562313 | Flow Cytometry Analysis |
| NK1.1 | PE-CF594 | BD Biosciences, 562864 | Flow Cytometry Analysis |
| Ly6G | PE-CF594 | BD Biosciences, 562700 | Flow Cytometry Analysis |
| CD19 | AF700 | BD Biosciences, 557958 | Flow Compensation |
| Icam2 | - | BD Pharmingen, 553326, 1:500 | Primary (IF) |
| anti-Rat | AF488 | ThermoFisher, A21208, 1:500 | Secondary (IF) |
